# Characterization of Ultrasonic Cavitation Energy for Efficient Soil Pretreatment: A Novel Approach for Rapid Detection of Available Potassium and Phosphorus Using CE-C4D

**DOI:** 10.2174/0115701794387336250516091810

**Published:** 2025-05-26

**Authors:** Jun Gao, Wei Li, Jiaoe Li, Rujing Wang

**Affiliations:** 1 University of Science and Technology of China, Hefei, 230026, China;; 2 Institute of Intelligent Machine, Hefei Institutes of Physical Science, Chinese Academy of Sciences, Hefei, 230031, China

**Keywords:** CE-C4D, ultrasonic extraction, pure water, potassium, phosphorus, ultrasonic cavitation yield, ultrasonic extractor, pretreatment

## Abstract

**Background:**

Soil sample pretreatment is a critical step in soil analysis, often involving complex processes and the use of organic solvents, which can lead to environmental pollution and inefficiency. In this regard, traditional methods are time-consuming and prone to human error, rendering them unsuitable for large-scale soil surveys. To address these challenges, this study proposes a novel rapid soil pretreatment method employing a device that integrates ultrasonic extraction with pure water and capillary electrophoresis using capacitively coupled contactless conductivity detection (CE-C4D). This study aims to enhance the efficiency and accuracy of soil nutrient analysis while minimizing environmental impact.

**Methods:**

Ultrasonic extraction using pure water as a solvent, combined with CE-C4D detection, was employed for rapid and efficient analysis of soil nutrients, particularly potassium and phosphorus. Parameters influencing ultrasonic extraction, including frequency, treatment time, and the relationship between solution factors and cavitation yield, were optimized through both simulations and experiments. Cavitation yield was assessed using iodine release, conductivity measurement, and fluorescence analysis. Additionally, the sound pressure distribution within the extraction tube was simulated, and temperature changes during ultrasonic treatment were monitored. The pretreatment device and detection method were validated using standard soil samples.

**Results:**

The optimal ultrasonic extraction parameters were identified as a frequency of 1.7 MHz and a treatment time of 10 minutes, resulting in the most effective cavitation yield. The use of pure water as a solvent improved both the efficiency of soil pretreatment and the environmental sustainability of the process. The CE-C4D system successfully detected available potassium and phosphorus in standard soil samples with high accuracy, showing trends consistent with standard values and relative standard deviations (RSD, n=4) of less than 5%. Blind tests on soil samples also demonstrated minimal error, with RSD (n=4) below 4%.

**Conclusion:**

This study presents a portable and efficient ultrasonic extraction device for soil pretreatment coupled with a custom-designed CE-C4D detector for the rapid analysis of available potassium and phosphorus. The use of pure water as a solvent significantly reduces environmental impact, simplifies the process, and enhances testing efficiency, reducing the required testing time from 15-20 days to within 30 minutes. This innovation provides a reliable solution for on-site soil analysis, offering potential applications in large-scale soil surveys and routine soil testing.

## 1 INTRODUCTION

Standardized soil testing pretreatment generally includes processes, such as sampling, air drying, grinding and sieving, digestion or extraction, and filtration or centrifugation [1]. Current methodologies for detection encompass preprocessing techniques tailored for laboratory settings [2]. Analysis of soil samples frequently employs extraction procedures, such as the Olsen method, Bray-1 method, Mehlich-3 method [3, 4], and the rapid ASI composite extraction method [5], subsequently utilizing the molybdenum-antimony anti-colorimetric technique for quantification [6]. Due to its lengthy detection cycle and complex operation, the method is not suitable for rapid, low-cost testing directly in farmland environments [7]. Other techniques, such as Inductively Coupled Plasma Atomic Emission Spectrometry (ICP-AES) [8], Atomic Absorption Spectrometry (AAS) [9], and X-ray Fluorescence Spectrometry (XRF) [10], are subject to limitations, including the necessity for intricate digestion procedures during sample pretreatment, interference of soil particle size on electrode-based analyses, and prerequisite of homogeneous particle distribution for sample compaction [11]. The digestion and extraction processes often require the use of strong acids, strong bases, or organic solvents for extraction, which easily pollutes the environment. Moreover, they are time-consuming and labor-intensive and have complicated operations that can introduce errors [12-14]. They are also complex, rely on many auxiliary equipment, and have high testing costs [15, 16].

 Modern agricultural production, largely driven by human activities, has significantly contributed to the increased environmental burden, particularly through carbon emissions associated with the transportation of agricultural products [17]. Sample collection alone requires hours to days, depending on batch size and site accessibility, followed by 1 to 3 days for transportation to laboratories under controlled conditions [18, 19]. Subsequent pretreatment stages introduce significant delays, *i.e*., air-drying fresh samples under ambient laboratory conditions require 7-10 days [18] to stabilize moisture content, while mechanical grinding (30-60 minutes) and sieving (30-40 minutes) further prolong preparation [20]. Component extraction *via* chemical digestion methods demands 1-2 hours per sample, often constrained by reagent-specific protocols and equipment availability [21]. While contemporary analytical instrumentation facilitates extract analysis within 1-20 minutes, the extended pre-analytical phase spanning multiple weeks from initial field sampling to final data interpretation remains constrained by batch-handling paradigms that prioritize laboratory operational efficiency at the expense of temporal resolution [22]. This systemic delay critically undermines data timeliness, particularly for early-collected samples awaiting batch pretreatment and analysis, rendering conventional methods inadequate for applications requiring rapid decision-making [23]. Consequently, field-deployable solutions integrating *in situ* sampling, accelerated pretreatment (*e.g*., portable grinding tools, ultrasonic extraction), and real-time on-site detection technologies are imperative to circumvent transportation bottlenecks and preserve the diagnostic value of time-sensitive soil health assessments.

 To effectively address this issue, efficient methods for rapid and accurate soil nutrient assessment must be developed. Portable detection systems have garnered considerable attention, especially in agricultural and food safety applications [24]. However, the soil pretreatment process remains challenging, as concurrently achieving high accuracy, convenience, safety, and environmental sustainability remains difficult [25]. Some research works have explored pure-water extraction, while others investigate ultrasonic wave soil pretreatment [26]. Despite advancements, few devices can integrate into portable systems for on-site agricultural use.

Ultrasonic waves, mechanical vibrations above 20 kHz, can propagate through gases, liquids, and solids. They possess unique properties, such as penetration, strong directionality, and the generation of microbubbles and local high temperatures under specific conditions [27]. In soil pretreatment, ultrasonic waves improve efficiency, reduce chemical reagents, have mild conditions, are eco-friendly, have time and cost benefits, enable easy automation, have wide applicability, and improve analysis accuracy [28, 29]. Ultrasonic waves accelerate the extraction process of chemical substances through the phenomenon of acoustic cavitation [30], which can significantly improve efficiency, reduce the dependence on chemical solvents, and lessen the environmental burden [31, 32]. They usually operate under normal temperature and pressure, which helps to protect the integrity of heat-sensitive components while saving time and cost [33]. In addition, ultrasonic equipment makes it easy to integrate automated systems for precise control and consistency, and it is applicable to the extraction of multiple soil types and target substances. Ultimately, reducing interfering substances can improve the accuracy and reliability of subsequent analysis [34].

For a long time, researchers have been using the electrical power of ultrasonic transducers to quantify the energy of ultrasonic waves acting on extraction objects [35, 36]. However, this method cannot reflect the amount of energy involved in soil extraction. The sound-induced free radicals from ultrasonic cavitation enable more accurate characterization of ultrasonic extraction energy by measuring cavitation yield [37, 38]. The cavitation yield can be detected through three methods: the iodine release method, conductivity method, and fluorescence method. This study explored the impacts of solution concentration, ultrasonic time, and frequency on cavitation yield. We simulated the sound pressure distribution and effective ultrasonic distance in the extraction tube, as well as the steps of ultrasonic extraction, CE-C4D parameters for detecting rapidly available potassium and available phosphorus in the soil, and the composition of the buffer solution. Standard soil samples, including Liaoning Brown Soil, Henan Yellow Tide Soil, Sichuan Purple Soil, Heilongjiang Black Soil, Xinjiang Gray Calcisols, Shaanxi Yellow Loess Soil, and Anhui Tidal Soil, were used as standard soil samples to verify the pretreatment device and detection method. Moreover, we verified the accuracy of ultrasonic extraction in detecting rapidly available potassium and phosphorus in soil through blind tests. These efforts contribute to the refinement of our existing rapid pretreatment module and improve the efficiency of equipment and methods for on-site rapid soil nutrient element pretreatment.

## 2 MATERIALS AND METHODS

### 2.1 Principle of Ultrasonic Cavitation

Ultrasonic cavitation is a phenomenon that occurs during the propagation of ultrasonic waves in a liquid [39]. The high-frequency vibration energy generated by the frequency and amplitude of ultrasonic waves leads to the formation of tiny bubbles or cavitation bubbles within the liquid [40]. These bubbles grow rapidly under the influence of ultrasonic waves and suddenly collapse [41] when the pressure decreases, creating an environment of high temperature and high pressure. This collapse triggers a series of physical and chemical effects [42]. This process can generate free radicals and strong oxidizing conditions in the liquid, thus initiating chemical reactions in the liquid, a phenomenon known as sonochemistry [43, 44]. Ultrasonic cavitation reactions do not stem from the direct interaction between sound waves and substance molecules. In liquids, the commonly used sound wave wavelengths range from 10 cm to 0.015 cm (corresponding to 15 kHz to 10 MHz), which are far greater than the molecular scale [45]. Ultrasonic cavitation is an effective method for promoting chemical reactions and increasing yields and is characterized by high atom economy and green reaction conditions [46]. Ultrasonic cavitation reactions primarily originate from acoustic cavitation, *i.e*, the formation, oscillation, growth, contraction, and collapse of bubbles in the liquid, as well as the physical and chemical changes it induces [47, 48]. The process of liquid acoustic cavitation involves concentrating the sound field energy and releasing it rapidly. When a cavitation bubble collapses, it generates a high temperature above 5000 K and a high pressure of approximately 5 × 10⁷ Pa within an extremely short time and small space, with a temperature change rate as high as 10⁹ K/s, accompanied by strong shock waves with a speed of up to 400 km/h [49, 50]. The rapidly collapsing small bubbles, due to their extremely short collapse time, cannot dissipate the heat generated by the compressed gas inside the bubbles in time, causing the temperature inside the bubbles to reach several thousand degrees and a high pressure of dozens of MPa. This phenomenon is known as the "cavitation effect" [51].

In 1910, Rayleigh established an equation describing the cavitation effect, which is referred to as the Rayleigh equation [52]. By solving the Rayleigh equation, an expression for the temperature inside the cavitation bubble when cavitation occurs can be approximately obtained, which is as follows:


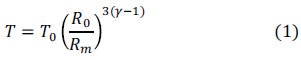


In the above equation (1), *T* is the high temperature inside the cavitation bubble after cavitation occurs; *T_0_* is the temperature of the liquid; *R_0_* is the initial radius of the cavitation bubble; *R_m_* is the radius of the cavitation bubble when cavitation terminates, and 𝛾 is the specific heat capacity ratio of the gas inside the bubble. If it is air, this value is approximately 1.34 [53]. A simple estimation using equation (1) shows that the temperature inside the cavitation bubble will not be lower than several thousand degrees when cavitation terminates. Such a high temperature directly causes the thermal decomposition of the gas inside the bubble and the medium at the gas-liquid interface, generating a large number of free radicals. These highly active free radicals enter the solution and trigger a series of secondary chemical reactions. This is the reason why sound can trigger chemical reactions in the liquid [54, 55].

The influence of sound intensity on ultrasonic cavitation reactions was established as early as the 1970s [39]. In the sound field of continuous sound waves, the critical sound intensity required to trigger ultrasonic cavitation reactions is no less than 0.7 W/cm². When using a modulated pulsed sound wave signal in a narrow-pulse sound field with a pulse width in the microsecond range, the critical value of sound intensity for triggering ultrasonic cavitation reactions drops to 0.3 W/cm². The loss of sound energy in the solution is related to the pulse width of the pulsed sound wave. The larger the pulse width, the greater the loss of sound energy in the solution, and vice versa. The appearance of the pulsed "cavitation peak" in the microsecond narrow-pulse range can reduce the loss of sound energy by about 50% compared to its appearance in the millisecond range [53]. Experiments have demonstrated that, macroscopically, ultrasonic cavitation reactions belong to zero-order reactions. In other words, the macroscopic reaction rate constant 𝐾 of ultrasonic cavitation reactions is independent of the initial concentration of compounds in the aqueous solution. The ultrasonic cavitation reaction rate is only a function of acoustic parameters, such as sound intensity. Moreover, at low sound intensity , there is a linear relationship between the reaction rate constant *K* and the sound intensity *I*. This conclusion is significant for the control of ultrasonic cavitation reactions and the design of large-scale ultrasonic cavitation reactors. From the perspective of the macroscopic characteristics of the cavitation effect, this is quite understandable. Once the sound intensity and the sound source frequency are preset, the cavitation effect in the solution is solely dependent on the number of cavitation bubbles, and the number of cavitation bubbles is the only factor that determines the level of ultrasonic cavitation yield in the solution [53].

### 2.2 Ultrasonic Cavitation Energy Measurement Method

Methods for measuring ultrasonic cavitation energy include acoustic intensity measurement (such as the radiation force method and thermistor method) [56], acoustic methods (acoustic attenuation method and acoustic impedance analysis) [57], optical methods (high-speed photography, light scattering techniques, and laser interferometry) [58], chemical methods (ultrasonic cavitation yield and chemical indicators) [59], physical methods (pressure sensors and particle image velocimetry) [56], electrical methods (conductivity measurement) [60], thermal methods (temperature measurement), and mechanical effect measurement (mass loss method and mechanical sensors) [61]. When using ultrasonic extraction for soil nutrient solutions in soil pretreatment, it is relatively convenient to adopt the ultrasonic cavitation yield measurement method.

The chemical titration method, which determines oxidants or acid-base products through titration, has a simple operation. The electrochemical method, which detects products through electrode signals, has high sensitivity. In the experiment, *KI* was used as a reaction probe. Under the action of ultrasound on the pure water solution, hydroxyl radicals were generated, and I⁻ ions were formed. The amount of *I₂* generated was determined by titration with a standard reagent sodium thiosulfate (*Na₂S₂O₃*) solution. Then, the number of hydroxyl radicals was calculated to reflect the ultrasonic cavitation yield.

### 2.3 Mechanism of Sonically-Induced Free Radical Reactions

Research on the "hot spot" theory of ultrasonic cavitation reveals that under the action of continuous sound waves or pulsed sound waves [62], cavitation bubbles generated by ultrasound in a liquid environment will, at the moment when the cavitation bubbles burst, cause the substances at the liquid-gas phase interface inside and around the cavitation bubbles to break into free radicals due to the extremely high temperature, pressure, and jet shock waves around the "hot spots" [63]. The basis for quantitatively detecting ultrasonic cavitation yield by the iodine release method, the conductivity method, and the fluorescence method also relies on a series of reactions initiated by free radicals. Ultrasonic cavitation yield is indirectly characterized by detecting these chemical reactions [64].

The iodine release method [65] works as follows: After potassium iodide (*KI*) solution containing a certain amount of dissolved air is subjected to the action of ultrasound, iodide ions will form iodine molecules and precipitate out. If a small amount of carbon tetrachloride (*CCl*_4_) is added to the solution, the effect will be better. If a small amount of starch is added to the solution, iodine will turn blue upon reacting with the starch. Then, the solution is titrated with sodium thiosulfate (*Na*_2_*S*_2_*O*_3_) solution. When the titration is completed, the solution returns to colorless. In this way, the amount of iodine molecules released can be determined by the consumption of sodium thiosulfate (*Na*_2_*S*_2_*O*_3_) during titration, and this amount can be regarded as the ultrasonic cavitation yield.

Sound-induced free radicals react with nitrogen and oxygen dissolved in water to generate nitric acid and nitrous acid, causing changes in the electrical conductivity of the solution [43]. This change in electrical conductivity is known as the ultrasonic cavitation yield measured by the conductivity method. Sound-induced free radicals also react with terephthalic acid, which has fluorescence activity, to produce hydroxylated terephthalic acid. The fluorescence absorption peak intensity of this reaction is used to quantify the ultrasonic cavitation yield by the fluorescence method (Fig. **1**) [66].

## 3 TEST AND ANALYSIS

### 3.1 Sound Pressure Simulation of the Piezoelectric Transducer Wafer in an Ultrasonic Extraction Tube

A classic COMSOL model, the water-roll support-air model, was developed using COMSOL Multiphysics^®^ software to analyze sound pressure distribution in water when an ultrasonic piezoelectric transducer wafer was operated in an ultrasonic extraction tube. The simulation considered a piezoelectric wafer with a thickness of 1 mm and a diameter of 10 mm, operating at a frequency of 1 MHz. The water medium was divided into 80 equal segments for the analysis. The piezoelectric transducer wafer was further divided into 20×2, 50×5, 100×10, and 640×64 equal parts to assess sound pressure distribution under different grid densities. Grid independence was verified by calculating the model using different grid densities and comparing simulation results under various grids. Similar outcomes indicated minimal model dependence on the grid, showing good convergence and reliable results (Fig. **2**).

(Quantitative detection of ultrasonic cavitation yield by the iodine release method, conductivity method, and fluorescence method).

By examining the sound pressure distribution at various axial distances along the pipe and at different depths within the liquid under different liquid surface heights, one can gain insight into how sound pressure is distributed throughout the liquid, as well as at the interfaces between the liquid and the pipe wall, and between the liquid and the air. From a simulation analysis perspective, under different load intensities, the sound pressure distribution in the pipe exhibits multiple focal points. The piezoelectric wafer primarily vibrates longitudinally, with some lateral vibration. The sound pressure distribution is the most concentrated at a distance of 5-6mm from the piezoelectric wafer, which is the most effective distance for ultrasonic cavitation (Fig. **3**).

### 3.2 Ultrasonic Extractor

The ultrasonic extractor consists of several key components, including the ultrasonic transducer wafer (operating at 1.7 MHz/36 V/18 W, 2 MHz/36 V/18 W, or 2.4 MHz/24 V/12 W), which is fixed and sealed with silica gel. A glass extraction tube (with an inner diameter of 150 mm) is also present and equipped with a cooling interlayer; cooling water flows upward from the bottom and circulates in the glass interlayer to cool the inside of the tube. The inlet and outlet of ultrapure water are located above the ultrasonic wafer on the left and right sides, respectively, near the extraction tube. Solid soil enters through the inlet at the top (Fig. **4**).

The electromechanical equivalent circuit of the ultrasonic transducer driving circuit is a circuit model that performs equivalent conversion of the mechanical vibration characteristics and electrical characteristics of the ultrasonic transducer. The design of this circuit is shown in Fig. (**5**), with the following parameters: dynamic resistance R_1_=9.8405 Ω, dynamic inductance L_1_=218.023 μH, dynamic capacitance C_1_=107.638 pF, static capacitance C_0_=2.24815 nF, quality factor Q=144.627, series resonant frequency F_s_=1.03893 MHz, and parallel resonant frequency F_P_=1.07575 MHz.

The electromechanical equivalent circuit of the ultrasonic transducer driving circuit (Fig. **5**) models the interdependence of mechanical vibration characteristics and electrical behavior through lumped parameters. These parameters govern energy conversion efficiency, resonant dynamics, and, ultimately, cavitation performance. The following are the key components:

**Dynamic resistance** (R_1_=9.8405Ω): It represents mechanical energy dissipation through viscous losses and internal friction. Lower R_1_ minimizes Joule heating, enhancing electroacoustic efficiency and sustaining stable cavitation intensity [67].

**Dynamic inductance** (L_1_=218.023 μH) and **dynamic capacitance** (C_1_=107.638 pF): They define the mechanical resonance frequency (Fs=1.03893MHz). Operation near this frequency maximizes mechanical displacement amplitude, which is critical for generating cavitation-inducing pressure gradients [68].

**Static capacitance** (C_0_=2.24815 nF): It reflects capacitive coupling between piezoelectric elements. Its impedance Z_C0_=1/(2πfC_0_) dominates off-resonance, necessitating impedance matching with the driving circuit to optimize power transfer [69, 70].

**Quality factor** (Q=144.627): It quantifies the energy storage-to-dissipation ratio. High Q ensures narrow resonance bandwidth (∆f=f_s_/Q≈7.18kHz), enabling precise frequency control to maintain cavitation thresholds, though requiring stricter thermal management (condensate water circulation cooling) [71].

The driving circuit employs a 1.05 MHz AC signal (18-20 *Vpp***)** superimposed on a 20-25V DC bias, strategically positioned between the series (F_s_=1.03893MHz) and parallel (F_P_=1.07575 MHz) resonances. This configuration exploits the transducer’s inductive region to compensate for C_0_-induced phase lag, ensuring an optimal power factor. The selected frequency lies within the Q-governed resonance bandwidth, amplifying piezoelectric strain to generate sufficient acoustic pressure for cavitation inception. Excessive deviation from F_s_ would elevate impedance, reducing mechanical output and cavitation efficacy.

The power supply adopts a direct current power source with a voltage range of 20-25V. The signal generator provides an alternating current signal in the form of a sine wave with a peak-to-peak voltage of 18-20Vpp and a frequency of 1.05MHz.

### 3.3 Ultrasonic Extraction Pretreatment Process

The operating steps of the ultrasonic extractor are as follows:

1. Place the cleaned and dried extraction tube on the test bench to stand and cool down for standby use.

2. Connect the inlet and outlet of the circulating cooling water and turn on the circulating cooling machine.

3. Connect the channels of the ultrapure-water inlet and outlet pipes. Introduce ultrapure water through the inlet to clean the ultrasonic transducer chip and the inner tube three times, with 10 mL each time.

4. Add soil samples through the solid sample inlet.

5. Add a certain amount of ultrapure water through the ultrapure water inlet.

6. Set the driving voltage and parameters of the signal source and turn on the power supply of the driving circuit.

7. Conduct ultrasonic extraction for a certain period of time.

8. Turn off the driving power supply, wait for the soil solids to settle, suck out the supernatant through the ultrapure-water outlet, and turn off the cooling water. Repeat step 3 to prepare for the next experiment.

#### 3.3.1 Soil Pretreatment Process

The soil pretreatment process is described in detail previously [72]. First, fresh soil samples are added to a soil rapid moisture detector to determine the soil water content (A). The soil is then weighed using a portable weighing instrument to obtain its weight (B). Afterward, the soil is placed into an ultrasonic extractor, and an appropriate amount of ultrapure water (C) is added. Once the ultrasonic extraction is completed, the resulting soil solution is filtered through a 3D-printed soil solution rapid filter, which has a filter cake pore diameter of 0.45 μm. The obtained soil solution can be directly injected into the CE-C4D instrument.

The schematic diagram of the soil pretreatment equipment and process is shown in (Fig. **6**), and it is referred to as the SSQP-IIM-115-PRO soil pretreatment device.

Soil nutrient element content *P*(*mg/kg*) is denoted by Eq. (2).


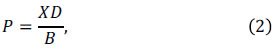


Where X(mg/L) is the detection value of the electrophoresis instrument; D(mL) is the sum of the solution volumes of A (mL) and C (mL), where A is the volume of fresh soil moisture content, and C is the volume of ultrapure water; and B(g) is the fresh soil mass.

### 3.4 CE-C4D Parameters

 The capillary specifications used in the analysis were 45 cm in total length with an inner diameter of 50 μm, and an effective length (Leff) of 40 cm. For potassium ion detection, the separation was carried out under a positive high voltage of +14 kV. Electrokinetic injection was performed using a positive high voltage of +10 kV for an injection time of 10 seconds. For phosphate ion detection, a negative high voltage of –14 kV was applied for separation, with electrokinetic injection at –12 kV and an injection time of 10 seconds. The soil extract used in the analysis was obtained by ultrasonic extraction of soil with ultrapure water (resistivity of 18.2 MΩ·cm) [72].

### 3.5 CE-C4D Buffer Solution System

To prepare the soil rapidly available potassium buffer solution (50× pTAE buffer), 3.03 g of Tris (hydroxymethyl) aminomethane and 0.18 g of ethylenediaminetetraacetic acid (EDTA) were added to a 50 mL volumetric flask. Ultrapure water (resistivity of 18.2 MΩ·cm) was used to fully dissolve the components. Then, 0.30 mL of glacial acetic acid was added, and the solution was diluted to 50 mL with ultrapure water. For use, this stock solution was diluted 50-fold to obtain a 1× pTAE buffer consisting of 10 mM Tris, 2 mM acetic acid, and 0.25 mM EDTA.

The electrophoresis running buffer must be freshly prepared daily. To do this, 0.60 g of polyvinylpyrrolidone (PVP, average molecular weight M = 30,000) was first dissolved in a small volume of ultrapure water in a plastic beaker. The resulting solution was transferred to a 100 mL volumetric flask. Then, 2 mL of the 50× pTAE buffer was added, and the final volume was brought up to 100 mL with ultrapure water.

 For the soil-available phosphorus buffer solution, 0.27 g of hydroxypropyl-β-cyclodextrin (HP-β-CD) was added to a 100 mL volumetric flask and dissolved in ultrapure water (18.2MΩ•cm resistivity). The solution was then adjusted with 150 µL of glacial acetic acid and topped up to 100 mL with ultrapure water [72].

The reagents used in the experiment, including ammonium acetate, potassium nitrate, standard phosphate ions, and potassium dihydrogen phosphate reagent, were obtained from Aladdin Bio-Chem Technology Co., Ltd. They were diluted with ultrapure water and stored in plastic bottles.

### 3.6 Soil Samples

Standard soil samples included Liaoning Brown Soil, Henan Yellow Tide Soil, Sichuan Purple Soil, Heilongjiang Black Soil, Xinjiang Gray Calcisols, Shaanxi Yellow Loess Soil, and Anhui Tidal Soil. These were all soil standard materials accredited by the Institute of Geophysical and Geochemical Exploration, Chinese Academy of Geological Sciences, the soil standard material accreditation agency in China.

A blind test of soil samples was conducted using soil samples from Taihe County, Fuyang City, Anhui Province, China. The samples were collected according to the sampling points specified in the testing standards of NY/T 1121.7-2014, which is issued by the National Ministry of Agriculture.

### 3.7 CE-C4D Detection Process for Soil Rapidly Available Potassium and Phosphorus

The soil pretreatment device (SSQP-IIM-115-PRO) integrates an ultrasonic extractor with capacitively coupled contactless conductivity detection (C4D) equipment for capillary electrophoresis (CE-C4D), enabling rapid pretreatment and analysis of soil-available potassium and phosphorus. The specific electrophoresis detection process, illustrated in (Fig. **7**), utilizes the following key parameters: a soil-to-liquid ratio of 1:20; an ultrasonic extraction time of 10 minutes; and capillary electrophoresis parameters of 45 cm×50 μm i.d., Leff =40 cm.

## 4 RESULTS AND DISCUSSION

### 4.1 Impact of Ultrasonic Time on Temperature

To investigate temperature variation during ultrasonic wave action on pure water, we selected three ultrasonic frequency transducer wafers (1.7MHz/36V/18W, 2MHz/36V/ 18W, and 2.4MHz/24V/12W) to make ultrasonic extraction tubes. Over 60 minutes, we measured and recorded water temperature every 5 minutes. The three independent measurements were averaged and a graph showing temperature change over time was created (Fig. **8**).

This graph illustrates the variation in ultrapure-water temperature over time under the influence of three different ultrasonic frequencies: 1.7MHz/36V/18W, 2MHz/36V/18W, and 2.4MHz/24V/12W. Error bars, representing the standard error of the mean with a sample size of n=3, were included to indicate the precision of the measured temperature values. They provide an estimate of the uncertainty associated with the mean temperature at each time point for the respective ultrasonic frequencies. Ultrasonic waves act on pure water, converting mechanical energy into thermal energy. As shown in the graph, the temperature of pure water shows an upward trend with the increase in ultrasonic time. Different frequencies of ultrasonic waves result in different rates of temperature increase in pure water. For example, in the initial stage, the temperature of pure water increases more rapidly at ultrasonic frequencies of 2 MHz and 2.4 MHz, whereas the temperature rise is slower at 1.7 MHz. This suggests that ultrasonic frequency significantly influences the efficiency of heat generation. Furthermore, when the ultrasonic treatment duration is sufficiently long, the rate of temperature increase begins to plateau. This trend may result from a balance being reached between the heat generated by the ultrasonic waves and the heat dissipated by the medium.

### 4.2 Iodine Release Method for Measuring Ultrasonic Cavitation Yield

#### 4.2.1 Influence of Solution Concentration

In ultrasonic extraction of soil solution, the influence of solution concentration on ultrasonic cavitation yield is directly related to the applicability of the method for extracting soil nutrients using ultrasonic waves, especially for various soils with different elemental contents. Ultrasonic cavitation yield was detected using different concentrations of KI solution, with the results shown in Fig. (**9**).

The ultrasonic cavitation yield was measured using the iodine release method at frequencies of 1.7 MHz/36 V/18 W, 2 MHz/36 V/18 W, and 2.4 MHz/24 V/12 W, with KI concentrations of 0.2 mol/L, 0.6 mol/L, and 1 mol/L. The following conclusions were drawn: as the duration of ultrasonic treatment increased, the ultrasonic cavitation yield increased overall. At the same ultrasonic frequency, different KI concentrations affected the ultrasonic cavitation yield. Within 15 minutes of ultrasonic action, the ultrasonic cavitation yield increased linearly with an increase in solution concentration, with a steep slope at all three frequencies. Beyond 15 minutes, the ultrasonic cavitation yield showed a turning point (1.7 MHz) or a trend of stabilization without an increase (2 MHz and 2.4 MHz; between 20-25 minutes). The analysis of the reasons suggests that ultrasonic waves of different frequencies have different wavelengths and energy distributions in liquids. The higher frequency of 2.4 MHz has a more concentrated energy distribution, which is not conducive to the formation of cavitation nuclei and the development of cavitation bubbles, while 1.7 MHz is more favorable for the formation and collapse of cavitation bubbles under experimental conditions. In terms of the influence of KI concentration, at frequencies of 1.7 MHz and 2 MHz, as the KI concentration increases, the physical properties of the solution change, with a decrease in surface tension that facilitates the formation of cavitation bubbles. Although an increase in viscosity has an impact, it promotes an increase in cavitation yield.

#### 4.2.2 Impact of Ultrasonic Time and Frequency

The duration of ultrasonic treatment and the frequency of the ultrasonic transducer are important operational parameters for the ultrasonic extraction tube. The impact of these two parameters on the yield of ultrasonic cavitation is related to the composition of the final ultrasonic extraction system. Fig. **10** shows the test results to identify the optimal combination of parameters.

As shown in Fig. **10**, with the increase in ultrasonic treatment time, the ultrasonic cavitation yield showed an upward trend. At a frequency of 1.7 MHz, as the ultrasonic time increased from 0 minutes, the ultrasonic cavitation yield gradually rose due to the continuous action of ultrasonic waves on the solution, giving cavitation nuclei more opportunities to grow and collapse, thereby increasing the cavitation effect. Similarly, at frequencies of 2 MHz and 2.4 MHz, the ultrasonic cavitation yield also increased with an increase in ultrasonic time. Differences were observed in ultrasonic cavitation yield at different frequencies; under the same ultrasonic time, the cavitation yields produced by 1.7 MHz, 2 MHz, and 2.4 MHz were different. When the ultrasonic time was short, this difference became more pronounced; for example, at 5 minutes, there was a significant difference in ultrasonic cavitation yield across different frequencies because the ultrasonic frequency affected the formation and collapse process of cavitation nuclei. At a higher frequency of 2.4 MHz, under its rated voltage, current, and power conditions, cavitation nuclei may not have enough time to grow fully before collapsing, resulting in a relatively lower cavitation yield. The 1.7 MHz frequency was relatively lower, and under its rated conditions, the efficiency of cavitation nuclei formation might be lower, resulting in the cavitation yield not being the highest. The 2 MHz frequency, under the same KI solution concentration, may be within a range that was more favorable for the formation and collapse of cavitation nuclei, and it may produce a relatively higher ultrasonic cavitation yield at certain ultrasonic times. In summary, under the three frequencies, the longer the ultrasonic treatment time, the higher the ultrasonic cavitation yield. The optimal ultrasonic cavitation yield can be obtained at frequencies of 1.7 MHz and 2 MHz.

### 4.3 Detecting Ultrasonic Cavitation Yield by Conductivity Method

Freshly prepared ultrapure water was used with an ultrasonic transducer of frequencies 1.7 MHz/36 V/18 W, 2 MHz/36 V/18 W, and 2.4 MHz/24 V/12 W. The solution’s electrical conductivity was measured every 2 minutes within 0-20 min. The difference between measured conductivity and initial ultrapure-water conductivity was calculated as ΔS, corresponding to ultrasonic cavitation yield.

Fig. **11** shows the conductivity of ultrapure water versus ultrasonic treatment time at three frequencies, with error bars denoting the standard error of the mean (n=3). It has been observed that the conductivity of ultrapure water increased with the duration of ultrasonic treatment at three ultrasonic frequencies. The change in conductivity varied at different frequencies. At 1.7 MHz (36 V/18 W), the rate of increase in conductivity was relatively fast, rising sharply after ultrasonic treatment exceeded 10 minutes. At 2.0 MHz (36 V/18 W), the trend of conductivity increase was more stable but significant overall, starting to increase noticeably after 5 minutes of ultrasonic treatment. At 2.4 MHz (24 V/12 W), the rate of increase in conductivity was relatively slow but still showed a clear upward trend. This change in conductivity was due to the cavitation effect produced by the propagation of ultrasonic waves in water. During the formation, growth, and collapse of cavitation bubbles, local high temperatures, pressures, and shearing forces were generated, leading to the breaking of chemical bonds in water molecules and the production of hydrogen ions (H⁺) and hydroxide ions (OH⁻), thereby increasing the conductivity of the water. Moreover, different frequencies of ultrasonic waves produced different cavitation effects. At a lower frequency of 1.7 MHz, the cavitation bubbles were larger in size and produced higher energy upon collapse, having a more significant impact on the ionization of water molecules. At a higher frequency of 2.4 MHz, the cavitation bubbles were smaller in size and produced relatively lower energy upon collapse, having a relatively weaker impact on the ionization of water molecules, but conductivity still gradually increased over time. Additionally, ultrasonic waves may also cause the release of more ions from trace impurities or dissolved gases already present in the water, further promoting an increase in conductivity.

### 4.4 Fluorescence Absorption Spectroscopy for Detecting Ultrasonic Cavitation Yield

Ultrasonic transducers operating at frequencies of 1.7 MHz (36 V, 18 W), 2 MHz (36 V, 18 W), and 2.4 MHz (24 V, 12 W) were used. A 0.5 mmol/L terephthalic acid solution was prepared for the experiments. The ultrasonic treatment times were set to 1, 2, 5, 10, and 20 minutes. Samples were prepared and labeled, and the fluorescence absorption spectra were measured. For each dataset, the absorption peak values were recorded. The absorption peak values obtained from the ultrasonic samples at different frequencies were plotted against the ultrasonic cavitation time. Ultrasonic waves produced high-pressure and low-pressure regions in liquids, leading to the formation, growth, and rapid collapse of bubbles within the liquid. During cavitation, the rapid collapse of bubbles resulted in a sharp increase in local temperature and pressure, causing the decomposition of water molecules (H_2_O) and the production of hydrogen atoms (H·) and hydroxyl radicals (·OH). When the sonically induced radicals (such as ·OH) came into contact with terephthalic acid molecules, they reacted chemically to form hydroxyl-terephthalic acid, producing a fluorescence absorption peak at specific wavelengths.

As shown in (Fig. **12**), fluorescence intensity generally increased with longer ultrasonic treatment times because more significant ultrasonic cavitation effects occurred over longer durations, causing structural changes in solution molecules and promoting the production of fluorescent substances. At the same treatment duration, the fluorescence intensity at 1.7 MHz was usually higher than that at 2 MHz and 2.4 MHz. The comparatively larger cavitation bubbles and higher collapse energy at 1.7 MHz excited solution molecules better to fluoresce. In contrast, the smaller cavitation bubbles and lower collapse energy at 2.4 MHz weakly excited solution molecules. Although the fluorescence absorption intensity at 2 MHz was higher than that at 1.7 MHz after 10 minutes of ultrasonic cavitation, considering the comprehensive factors of increased system temperature with prolonged cavitation time, a balanced decision was made. Under a treatment time of 10 minutes, the frequency of 1.7 MHz, which exhibited the second-highest fluorescence absorption, was deemed more suitable for on-site soil pretreatment.

Based on experimental results detecting ultrasonic cavitation yield by the iodine release method, conductivity method, and fluorescence spectroscopy, the following conclusions were drawn: 1) cavitation yield increased with ultrasonic time and decreased with frequency; 2) among three ultrasonic transducers (1.7 MHz/36 V/18 W, 2 MHz/36 V/18 W, 2.4 MHz/24 V/12 W), the optimal transducer was 1.7 MHz/36 V/18 W, with a 10-minute ultrasonic time, for maximum cavitation yield, which was considered more suitable for on-site soil pretreatment; 3) condensing the extracted solution to cool the ultrasonic extraction tube maintained the ultrasonic temperature from rising, ensuring the feasibility of ultrasonic soil solution extraction.

### 4.5 CE-C4D Detection Process for Available Potassium and Phosphorus in Soil

#### 4.5.1 Standard Soil Samples Testing

The SSQP-IIM-115-PRO soil pretreatment device was used to process soil samples, and the CE-C4D detector was utilized to measure the rapidly available potassium and phosphorus. The detection targets were standard soil samples. The detection results are shown in Fig. (**13**).

Fig. (**13**) indicates a certain correlation between the detection values of standard soil samples after ultrasonic extraction pretreatment and the standard values of the samples before pretreatment. The results of standard soil testing showed that the relative standard deviations (RSD) (n=4) of the measured values of available potassium and phosphorus were both less than 5%. The detection values of available potassium in all soil samples followed the same trend as the standard values, except for the Henan Yellow Tide Soil. In the detection of phosphorus, the measured values of soil-available phosphorus generally followed the same pattern as the standard values, except in the case of Sichuan Purple Soil. This soil pretreatment and detection method can be applied to the rapidly available potassium and available phosphorus in various soil types. The data have high repeatability, and ultrapure water can be used as the soil extraction solvent in the field to avoid environmental pollution. Moreover, personnel do not require special training, which greatly enhances safety.

#### 4.5.2 Blind Soil Samples Testing

The SSQP-IIM-115-PRO soil pretreatment device and CE-C4D detector were utilized for feasibility validation in blinded soil samples. Laboratory test standard values were determined according to the National Ministry of Agriculture NY/T1121.7-2014 at sampling points. Ten independent soil samples were tested, with results depicted in Fig. (**14**).

As shown in (Fig. **14**), in the blind test, the detection values of the soil samples showed a consistent correlation with the standard values, indicating that the method is applicable for on-site soil sample pretreatment and detection.

The available potassium RSD (n=4) in soil samples was less than 4%, and phosphorus RSD (n=4) was also less than 4%, indicating high precision in detected values. The soil pretreatment method and the CE-C4D method employed demonstrated high reliability.

## STUDY LIMITATIONS

However, the current research has only been validated for the quantification of available potassium and phosphorus, using standard soil samples and blind test samples primarily from Anhui sandy loam soils. There remains a lack of systematic evaluation for the diverse soil types encountered in the national soil census. Additionally, while the portable battery-powered design meets the needs for portable operations, energy optimization for extended surveys remains a practical challenge. These limitations highlight prioritized directions for future research, including cross-regional validation and adaptive detector configurations for broader agrochemical analytes. While optimized for the quantification of available potassium and phosphorus, the modular architecture of the proposed system permits future expansion to other elements (*e.g*., nitrates) by expanding the detection scheme and system. Further studies should investigate longterm cavitation stability under field conditions and multilayered soil heterogeneity effects. Nevertheless, this study provides a paradigm shift toward sustainable precision agriculture, aligning with UN Sustainable Development Goals by enabling data-driven soil management while minimizing ecological footprints.

## CONCLUSION

This study focused on research into methods for portable, on-site soil pretreatment devices and detectors, utilizing ultrapure water as the soil extraction agent and ultrasonic extraction tubes as the rapid pretreatment device for soil extraction. The SSQP-IIM-115-PRO ultrasonic extraction pretreatment device for soil was constructed, and a self-designed and constructed CE-C4D detector was used as the detection equipment for available potassium and phosphorus in the soil samples. This study proposed an integrated methodology for rapid, eco-efficient soil nutrient analysis through the synergistic implementation of optimized ultrasonic extraction and portable CE-C4D detection. The SSQP-IIM-115-PRO ultrasonic pretreatment device, operating at a resonance-tuned frequency of 1.7 MHz with 10-minute extraction cycles, achieved maximized cavitation yields through multi-parametric optimization (frequency, temporal duration, and sound field homogeneity). The substitution of ultrapure water for conventional organic solvents eliminated secondary contamination risks while achieving comparable extraction efficiencies, which were validated by <4% RSD (n=4) in blind tests against certified soil reference materials.

The CE-C4D detection system exhibited robust analytical performance for rapidly available potassium and phosphorus quantification, with sensitivity thresholds meeting agronomic survey requirements. This electrochemical detection modality circumvented optical interference limitations inherent in turbid soil matrices, ensuring field-deployable accuracy. Notably, the methodology reduced total analysis time from 15-20 days (conventional protocols) to <30 minutes and a 99.8% temporal efficiency gain without compromising precision.

Technologically, the system’s innovation lies in its dual-environmental stewardship: (1) eliminating solvent-derived VOC emissions through aqueous-phase extraction and (2) enabling on-site analysis via lightweight, low-power hardware compatible with portable energy sources. Such features address critical gaps in large-scale soil surveys, particularly in remote or resource-limited regions without laboratory infrastructure.

## AUTHORS' CONTRIBUTIONS

The authors confirm their contribution to the paper as follows: data collection: JL; data analysis or interpretation: WL; methodology: RW; draft manuscript: JG. All authors reviewed the results and approved the final version of the manuscript.

## Figures and Tables

**Fig. (1):**
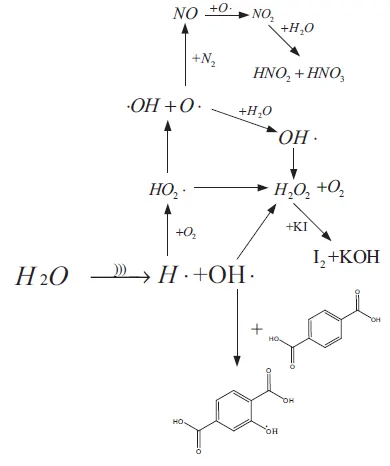
Mechanism of sonically-induced free radical reactions.

**Fig. (2):**
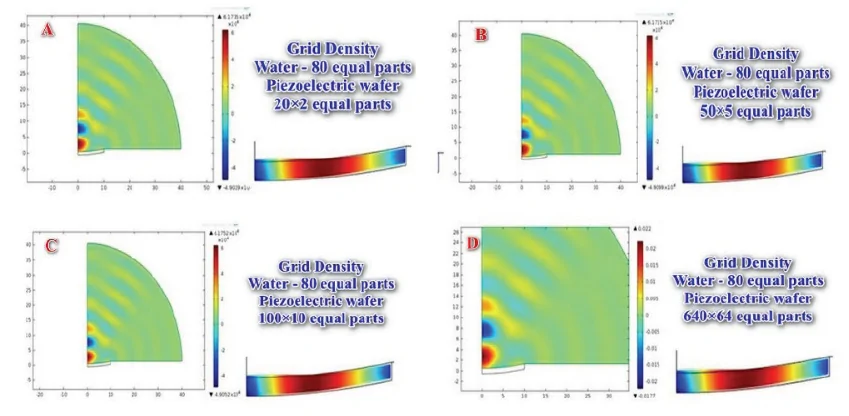
Water-roll support-air COMSOL model with different grid densities (piezoelectric wafer thickness of 1mm, diameter of 10mm, and frequency of 1MHz, with water divided into 80 equal parts, and piezoelectric wafer divided into A: 20×2 equal parts, B: 50×5 equal parts, C: 100×10 equal parts, and D: 640×64 equal parts).

**Fig. (3):**
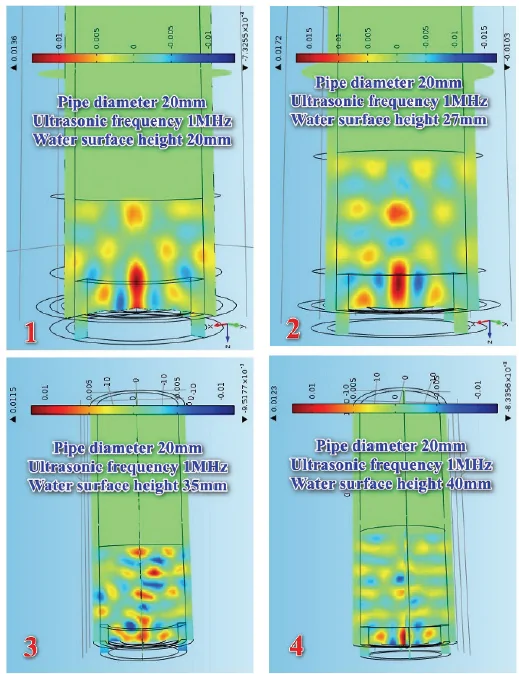
Ultrasonic vibration sound pressure distribution at different liquid levels (Water-roll support-air COMSOL model, liquid levels 1:20 mm; 2:27 mm; 3:35 mm; 4:40 mm).

**Fig. (4):**
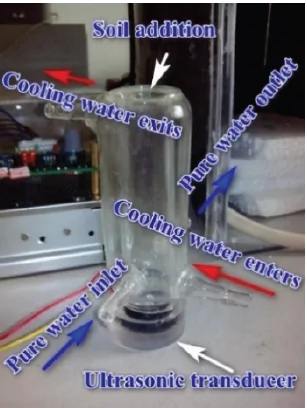
Ultrasonic extractor device component.

**Fig. (5):**
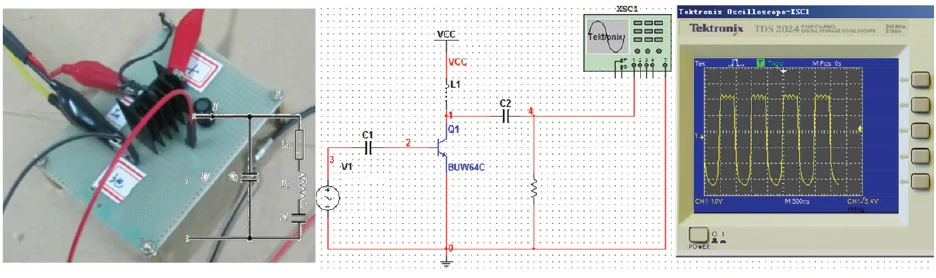
The electromechanical equivalent circuit of the ultrasonic transducer driving circuit (direct current power supply: 20-25 V. The alternating current signal: sine wave, peak-to-peak voltage 18-20 Vpp, and frequency 1.05 MHz).

**Fig. (6):**
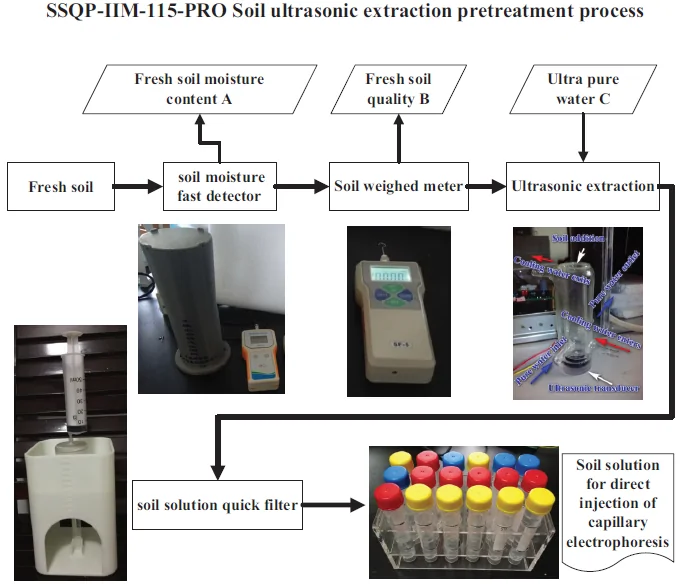
The equipment and flow chart of the ultrasonic extractor for soil pretreatment (SSQP-IIM-115-PRO).

**Fig. (7):**
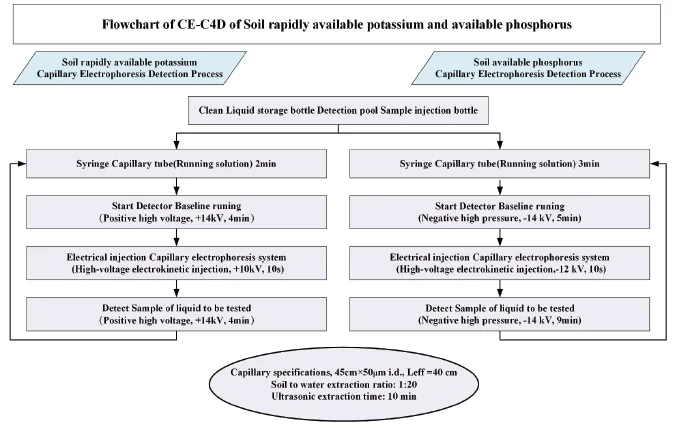
Flow diagram of CE-C4D of soil rapidly available potassium and available phosphorus.

**Fig. (8):**
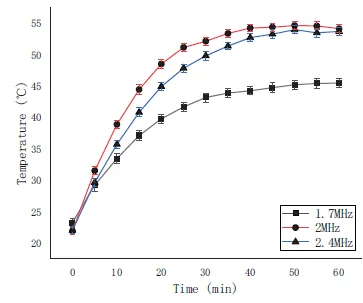
Graph of the change in ultrapure-water temperature over time under the action of three ultrasonic frequencies (1.7MHz/36V/18W, 2MHz/36V/18W, 2.4MHz/24V/12W; error bars indicate SEM (n=3)).

**Fig. (9):**
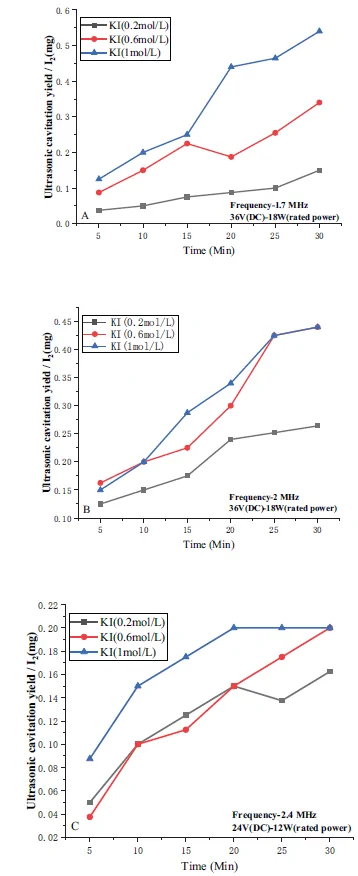
Iodine release method for measuring ultrasonic cavitation yield (Three frequencies: 1.7 MHz/36 V/18 W, 2 MHz/36 V/18 W, 2.4 MHz/24 V/12 W; KI concentrations are 0.2 mol/L, 0.6 mol/L, 1 mol/L, respectively).

**Fig. (10):**
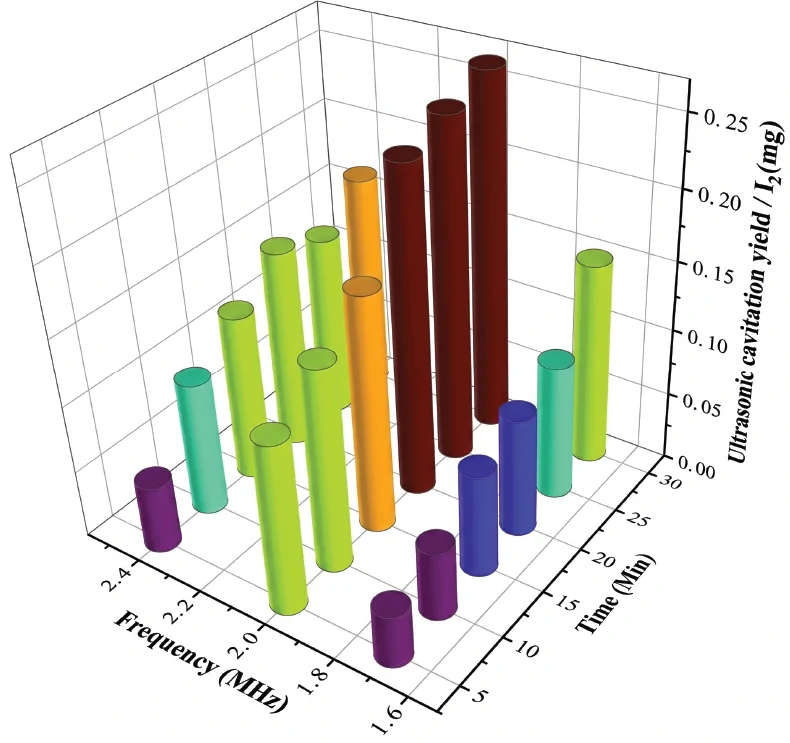
Comparison of ultrasonic cavitation yield from three ultrasonic transducers with different frequencies (1.7 MHz/36 V/18 W, 2 MHz/36 V/18 W, 2.4 MHz/24 V/12 W; KI, 0.2 mol/L) over varying ultrasonic treatment times.

**Fig. (11):**
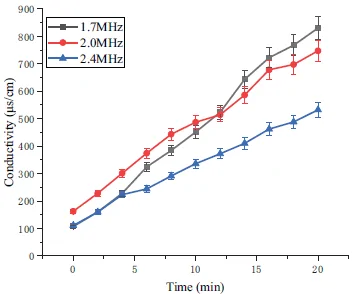
The conductivity of ultrapure water varies with ultrasonic treatment time at three frequencies. Error bars indicate SEM (n=3).

**Fig. (12):**
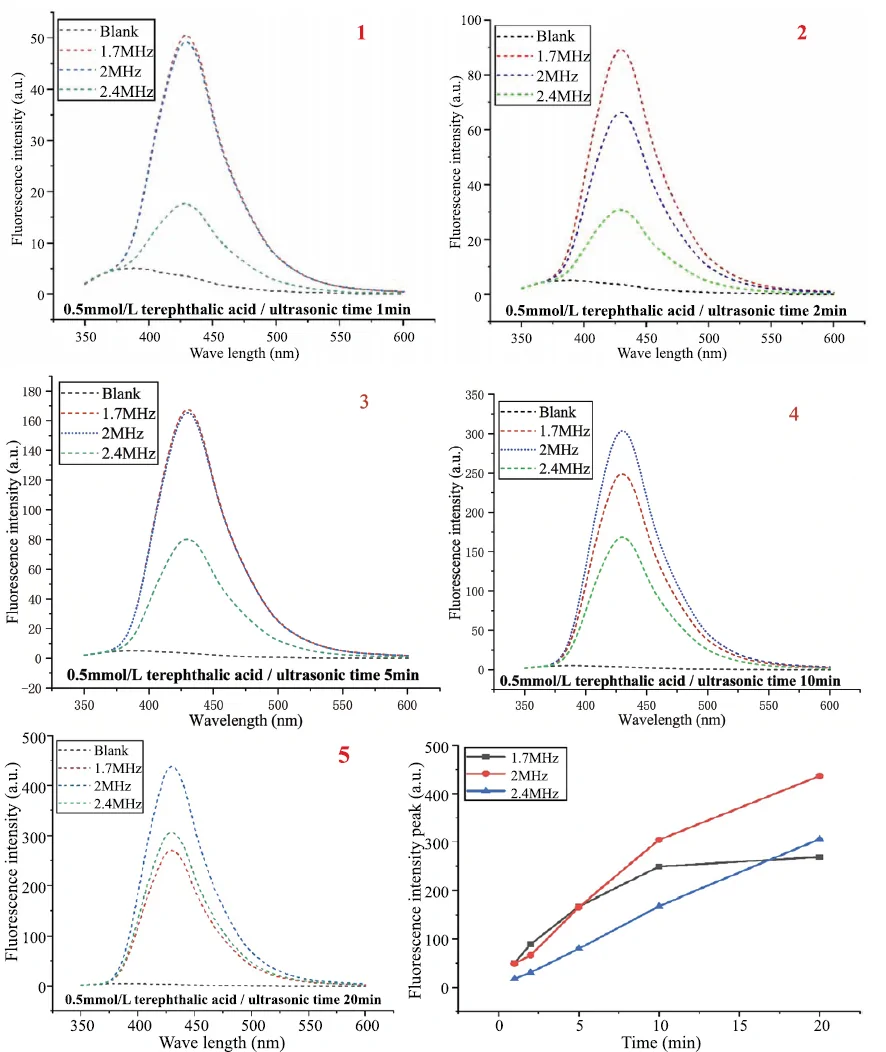
Fluorescence absorption spectroscopy was used to detect the yield of ultrasonic cavitation. The fluorescence absorption curves and fluorescence peak values at different ultrasonic times and frequencies (where the ultrasonic time was 1.1 min, 2.2 min, 3.5 min, 4.10 min, 5.20 min; and 0.5 mmol/L terephthalic acid).

**Fig. (13):**
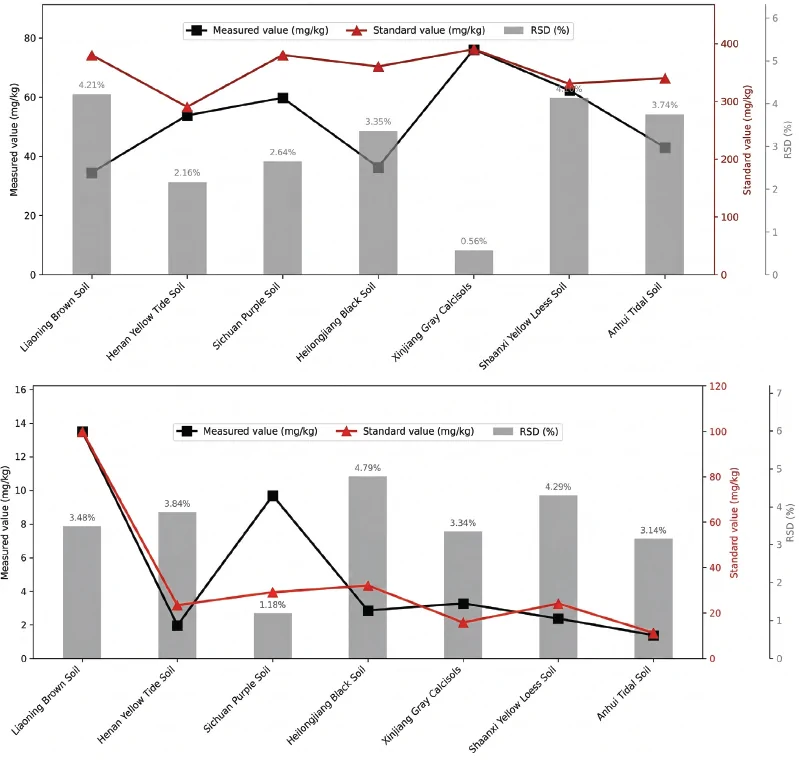
CE-C4D equipment for ultrasonic extraction of available potassium (upper panel) and phosphorus (lower panel). Comparison of measured values against standard values for standard soil samples, RSD (n=4).

**Fig. (14):**
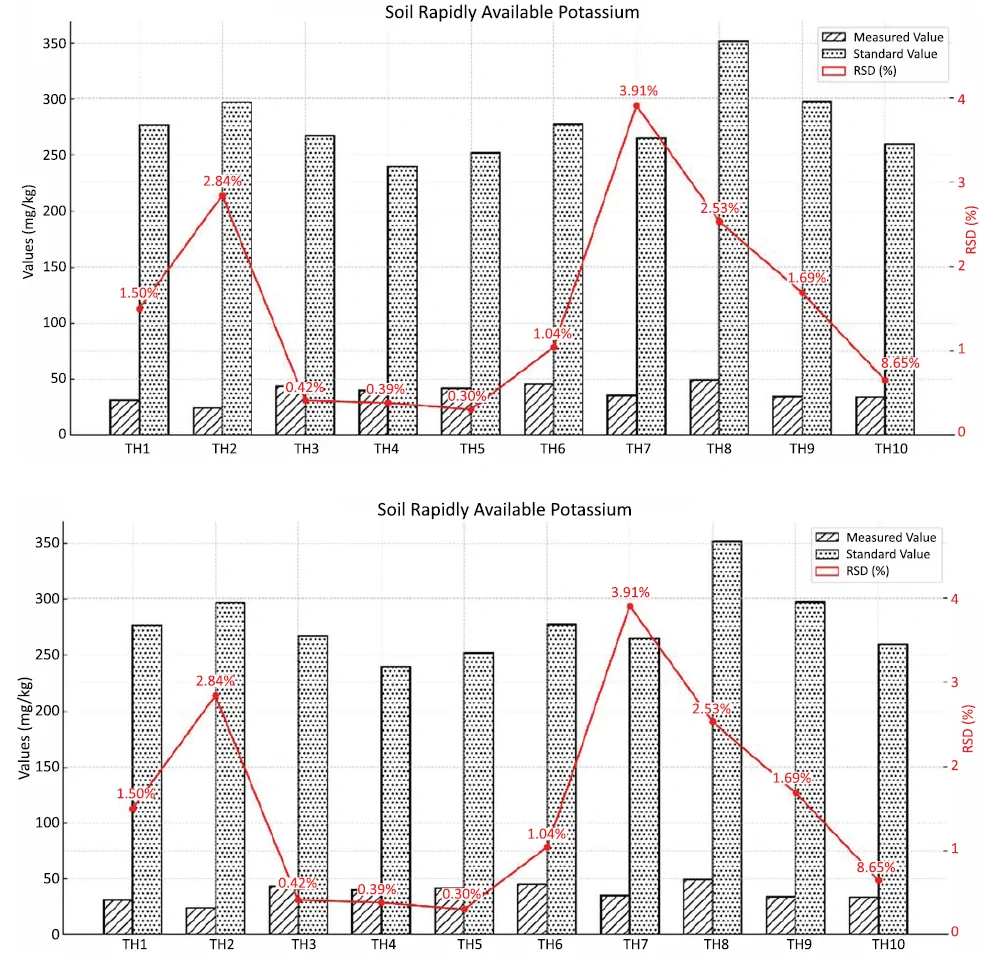
CE-C4D equipment for ultrasonic extraction of available potassium (upper panel) and phosphorus (lower panel) in soil samples. Comparison of measured values against standard values for the blind test conducted for soil samples, RSD (n=4) (Soil samples were collected from Taihe County, Fuyang City, Anhui Province, China).

## Data Availability

All the data and supporting information are provided within the article.

## LIST OF ABBREVIATIONS

1×pTAE Buffer: 10 mM Tris + 2 mM HAc + 0.25 mM EDTA
CE-C4D: Capillary Electrophoresis with Capacitively Coupled Contactless Conductivity Detection
EDTA: Ethylenediaminetetraacetic Acid
H: Hydrogen Atom
HAc: Acetic Acid
HP-β-CD: Hydroxypropyl-β-cyclodextrin
i.d.: Inner Diameter
I_2_: Elemental Iodine
L_eff_: Effective Length
Na_2_S_2_O_3_: Sodium Thiosulfate
OH: Hydroxyl Radical
PVP: Polyvinylpyrrolidone
RSD: Relative Standard Deviation
SSQP-IIM-115-PRO: System of Soil Quick Pretreatment-Institute of Intelligent Machine-115 Professional
Tris: Tris (hydroxymethyl) Aminomethane
